# A cross-sectional assessment of the diagnostic value of serum ceruloplasmin for Wilson’s disease in children

**DOI:** 10.1097/MD.0000000000048082

**Published:** 2026-03-13

**Authors:** Maysam Yaldany, Fares Abboud, Sultaneh Haddad, Suha Madanieh, Jaber Mahmod, Manar Alhazaimeh, Ghosoun Atoom

**Affiliations:** a Department of Gastroenterology, Children’s University Hospital, Damascus, Syria; b Faculty of Medicine, Damascus University, Damascus, Syrian Arab Republic; c Department of Pediatrics, Children’s University Hospital, Damascus, Syrian Arab Republic; d Department of Internal Medicine, Faculty of Medicine, Mu’tah University, Mu’tah, Jordan; e Department of Internal Medicine, Faculty of Medicine, Yarmouk University, Irbid, Jordan.

**Keywords:** ceruloplasmin, cutoff value, diagnostic accuracy, pediatrics, ROC curve, sensitivity, specificity, Wilson’s disease

## Abstract

Wilson’s disease (WD) is a rare autosomal recessive disorder of copper metabolism requiring early diagnosis to prevent severe hepatic and neurological damage, particularly in children, where diagnostic challenges are pronounced. Serum ceruloplasmin (CPN) is a critical biochemical marker, yet its diagnostic accuracy and optimal cutoff value in pediatric populations need further evaluation in specific regional contexts. This study evaluates the diagnostic performance of serum CPN in diagnosing WD among children at Damascus University Children’s Hospital and determines an optimal diagnostic cutoff value for this population. A bidirectional cross-sectional study was conducted from January 2019 to December 2022 at Damascus University Children’s Hospital, including 80 children diagnosed with WD (case group) and 80 children with hepatic symptoms but without WD (control group), all under 13 years. Serum CPN was measured using immunoturbidimetry. Data on demographics, clinical presentations, Kayser–Fleischer rings, CPN levels, and 24-hour urinary copper were collected. Statistical analyses included *t*-tests, receiver operating characteristic analysis, and multiple linear regression (Statistical Package for Social Sciences). Ethical approval and informed consent were obtained. Mean age in the WD group was 119.15 ± 30.47 months (57.5% female). Serum CPN was significantly lower in WD (9.8 ± 3.2 mg/dL) versus controls (27.6 ± 10.4 mg/dL, *P* < .001). Receiver operating characteristic analysis showed high diagnostic accuracy (AUC = 0.967, 95% CI: 0.945–0.988), with an optimal CPN cutoff of 15 mg/dL (sensitivity 93.8%, specificity 85.0%). Regression identified age, female sex, neurological symptoms, Kayser–Fleischer rings, and urinary copper as predictors of lower CPN. Serum CPN is a highly accurate diagnostic marker for pediatric WD, with a 15 mg/dL cutoff optimizing sensitivity and specificity. Interpretation should consider age, sex, and clinical features.

## 1. Introduction

Wilson’s disease (WD) is an autosomal recessive disorder caused by ATP7 B gene mutations, impairing copper metabolism and leading to accumulation in the liver and brain.^[1]^ With a prevalence of 1:30,000 to 1:100,000, WD can cause severe hepatic and neurological complications, particularly in children, where early diagnosis is critical.^[2]^ Pediatric diagnosis is challenging due to symptom overlap with other hepatic disorders and variability in biochemical markers.^[3]^

Serum ceruloplasmin (CPN), a copper-carrying glycoprotein, is a key diagnostic marker for WD due to its low levels in affected individuals.^[4]^ Its accuracy varies with age, sex, and clinical presentation, with reported sensitivity of 80% to 95% and specificity of 70% to 90% depending on the cutoff.^[5-8]^ In resource-limited settings, CPN is vital for screening, as genetic testing is often unavailable.^[9,10]^ This study, conducted at Damascus University Children’s Hospital, Syria, hypothesizes that CPN is a reliable diagnostic marker for pediatric WD and aims to determine an optimal cutoff and influencing factors to refine diagnostic algorithms.^[11,12]^

## 2. Materials and methods

### 2.1. Study design

This bidirectional cross-sectional study, conducted from January 1, 2019 to December 31, 2022, at Damascus University Children’s Hospital, adhered to STROBE guidelines.^[13]^ It included 80 children with confirmed WD (case group) and 80 age-matched children with hepatic symptoms but without WD (control group), all under 13 years.

### 2.2. Setting

The study was performed at a tertiary pediatric referral center in Damascus, Syria, serving a diverse population. Data were collected from hospital records and laboratory databases during routine clinical evaluations.

### 2.3. Participants

Eligible cases were children diagnosed with WD per EASL criteria using a composite reference standard that included clinical symptoms, 24-hour urinary copper excretion, Kayser–Fleischer rings, and, when available, ATP7B genotyping^[11]^; low serum CPN was considered supportive but not as a stand-alone diagnostic criterion. Controls had confirmed non-WD hepatic disorders (e.g., autoimmune hepatitis, viral hepatitis). Exclusion criteria included incomplete records, lack of consent, or conditions known to affect CPN (e.g., malnutrition).

### 2.4. Data sources/measurement

Serum CPN was measured using immunoturbidimetry (Roche Diagnostics), a standardized method.^[14]^ Urinary copper was quantified via atomic absorption spectroscopy. Kayser–Fleischer (KF) rings were assessed by slit-lamp examination. Data were extracted by trained staff, with double-entry to ensure accuracy.

### 2.5. Bias

Selection bias was minimized by consecutive sampling. Measurement bias was reduced through standardized protocols and calibrated equipment. Confounding was addressed via regression adjustment for age, sex, and clinical factors.

### 2.6. Sample size

A sample size of 160 (80 cases, 80 controls) was calculated to detect a 0.9 AUC with 80% power, 5% significance, assuming a 0.15 effect size based on prior studies.

### 2.7. Statistical methods

Descriptive statistics summarized demographics and clinical data. Independent *t*-tests compared CPN levels. Receiver operating characteristic analysis determined AUC and optimal CPN cutoff (Youden’s index, closest-to-top-left method). Sensitivity, specificity, positive predictive value, negative predictive value, LR+, and LR− were calculated. Multiple linear regression identified predictors of CPN levels. Analyses used Statistical Package for Social Sciences (v26), with *P* < .05 as significant. Missing data (<5%) were handled via listwise deletion. Distributional assumptions were evaluated using the Shapiro–Wilk test and visual inspection of histograms and Q–Q plots; as CPN levels were approximately normally distributed in both groups, we used independent *t*-tests to compare means.

## 3. Results

**Figure 1.**
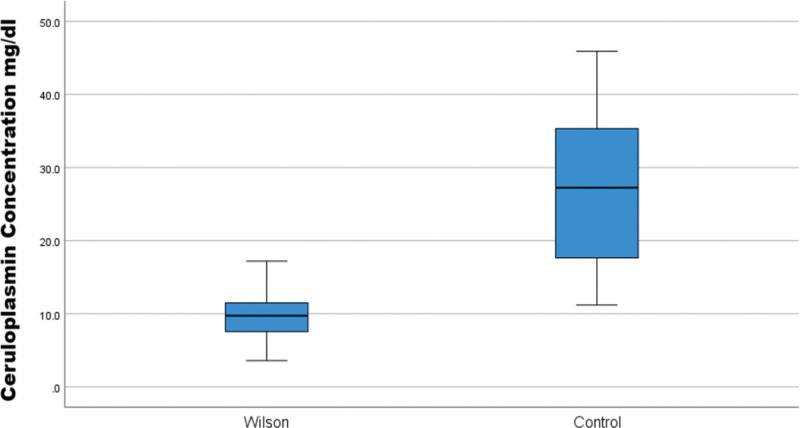
Distribution of serum ceruloplasmin levels in WD and control groups, highlighting significant separation. WD = Wilson disease.

**Figure 2.**
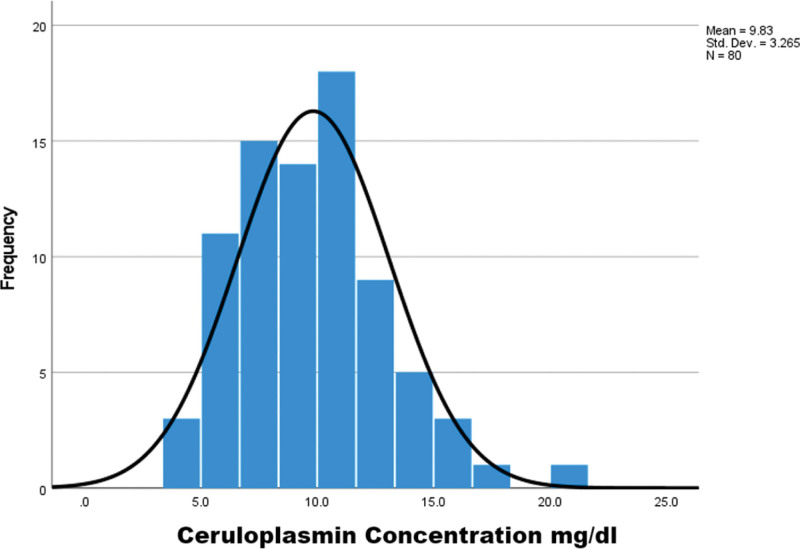
Ceruloplasmin concentration in WD patients. WD = Wilson disease.

**Figure 3.**
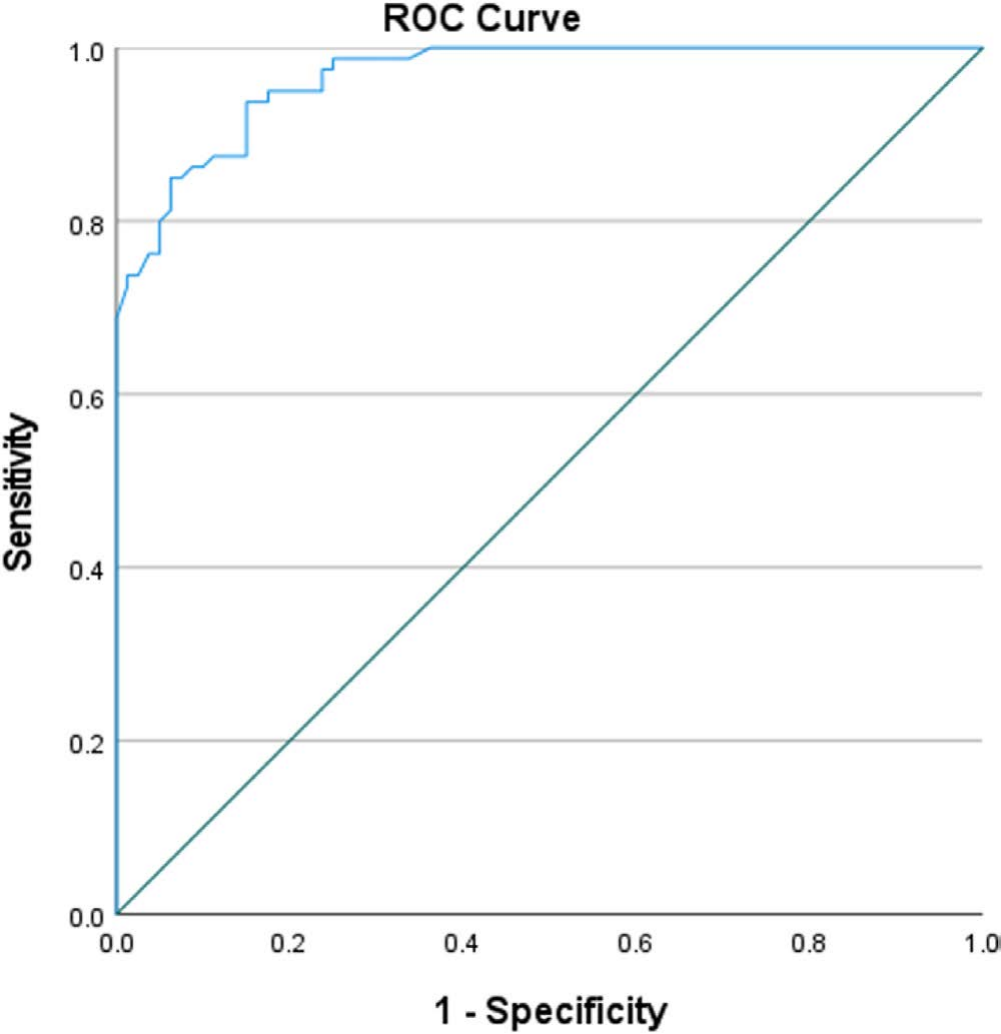
ROC curve demonstrating CPN’s discriminative ability. CPN = ceruloplasmin, ROC = receiver operating characteristic.

Of 160 children, the WD group (n = 80) had a mean age of 119.15 ± 30.47 months (57.5% female); controls (n = 80) were age-matched. Serum CPN was significantly lower in WD (9.8 ± 3.2 mg/dL) versus controls (27.6 ± 10.4 mg/dL, *P* < .001; Figs. 1 and 2). Receiver operating characteristic analysis yielded an AUC of 0.967 (95% CI: 0.945–0.988, *P* < .001), with an optimal cutoff of 15 mg/dL (sensitivity 93.8%, specificity 85.0%, positive predictive value 86.2%, negative predictive value 93.2%, LR+ 6.25, LR− 0.073; Fig. 3). Multiple regression identified significant predictors of lower CPN: age (β = 0.733, *P* < .001), female sex (β = −0.211, *P* = .041), neurological symptoms (β = −0.176, *P* = .040), KF rings (β = −0.193, *P* = .019), and urinary copper > 40 µg/24 h (β = −0.642, *P* = .033; Table 1).

**Table 1:** Comparison of diagnostic performance of serum ceruloplasmin across studies.

Study	Population	CPN cutoff (mg/dL)	Sensitivity (%)	Specificity (%)	AUC
Current study (2024)	Pediatric, Syria (n = 160)	15.0	93.8	85.0	0.967
Mak et al, 2008^[5]^	Mixed, Hong Kong (n = 126)	20.0	88.0	82.0	0.940
Merle et al, 2009^[6]^	Adult, Germany (n = 98)	20.0	90.0	88.0	0.950
Xu et al, 2018^[7]^	Mixed, China (n = 415)	14.6	92.0	83.5	0.936
Yang et al, 2022^[8]^	Pediatric, China (n = 287)	15.2	91.5	84.0	0.945

This table compares our findings with prior studies, showing our cutoff aligns closely with pediatric populations but differs from adult-focused studies.

AUC = area under the curve, CPN = ceruloplasmin.

## 4. Discussion

Serum CPN demonstrated excellent diagnostic performance for WD in this pediatric cohort, with an AUC of 0.967 (95% CI: 0.945–0.988), confirming its status as a highly discriminative biochemical marker. This aligns closely with prior large-scale evaluations reporting AUC ranges between 0.93 and 0.98 (5, 6, 8). The optimal cutoff identified in our study (15 mg/dL) provided both high sensitivity (93.8%) and specificity (85.0%), values comparable to those reported in major Asian pediatric cohorts, such as the 15.2 mg/dL threshold identified by Yang et al.^[8]^ These findings reinforce the clinical value of CPN as a first-line diagnostic test in settings where ATP7B genotyping is unavailable or cost-prohibitive.^[9,12]^

Our cutoff value was lower than the traditional adult threshold of 20 mg/dL described in earlier Western studies,^[5,6]^ underscoring the need for pediatric- and population-specific interpretation. Age-related hepatic maturation may partially explain why children exhibit slightly higher baseline CPN levels than infants but lower variability than adults.^[3]^ This is supported by our regression analysis, where younger age predicted lower CPN levels – a pattern described previously by Kim et al, who noted age-dependent biochemical variation in pediatric WD.^[3]^

Sex differences also emerged as a significant predictor, with female patients exhibiting lower CPN levels independent of disease severity. Ferenci et al similarly reported sex-associated phenotypic variations in WD, suggesting possible hormonal modulation of copper metabolism.^[13]^ Although biological mechanisms remain incompletely understood, this finding highlights the importance of incorporating sex into diagnostic algorithms.

Clinical severity markers – including neurological involvement and the presence of KF rings – were associated with lower CPN levels, consistent with the progression pattern of copper accumulation seen in advanced WD.^[11]^ Elevated 24-hour urinary copper excretion (>40 µg/24 hours) also correlated strongly with lower serum CPN. This association reflects the central defect of ATP7B-mediated copper excretion^[14]^ and aligns with the diagnostic framework endorsed by EASL and AASLD guidelines.^[11,12]^

When compared with the broader literature, our diagnostic metrics appear robust. The AUC observed in our population exceeded that reported by Mak et al (0.94) and was comparable to Merle et al (0.95).^[5,6]^ Sensitivity in our cohort (93.8%) surpassed values reported by Yang et al (91.5%) and was consistent with studies where pediatric patients were analyzed separately, supporting the notion that CPN decreases more uniformly in children with WD.^[8]^ Specificity, although slightly lower than in Merle et al, remains within clinically acceptable ranges and reflects the known overlap between WD and other chronic liver diseases, as previously highlighted by Cauza et al.^[4]^

Despite its strong diagnostic utility, serum CPN is not definitive when used in isolation. Low CPN may occur in protein-losing states, severe malnutrition, or other hepatic conditions, reiterating the need for multi-modal assessment including urinary copper, ophthalmologic evaluation, and clinical scoring systems.^[4,9,11]^ In resource-limited environments, however, the availability, affordability, and rapid turnaround of immunoturbidimetric CPN assays position them as an invaluable screening tool. Although enzymatic assays measuring oxidase activity offer higher specificity, their limited accessibility was similarly noted by Merle et al^[6]^ and remains a constraint in many low- and middle-income countries.

Emerging biomarkers such as relative exchangeable copper (REC) and exchangeable copper fraction (CuEXC) have demonstrated superior discriminative ability in recent European studies.^[14,15]^ Future investigations in Middle Eastern pediatric populations should evaluate whether incorporating such markers alongside CPN improves diagnostic accuracy, particularly in neurologically presenting cases.

This study’s strengths include the rigorous selection of age-matched controls, standardized laboratory methodology, and the use of regression modeling to identify independent predictors of CPN variation. However, limitations must be acknowledged. The single-center design may limit generalizability across genetically diverse populations. Genetic confirmation was unavailable for all cases due to cost barriers, though this reflects real-world diagnostic limitations in many regions. Finally, the cross-sectional nature of the study precludes assessment of CPN trends over time or during treatment, an area identified by Kasztelan-Szczerbinska & Cichoz-Lach as critical for future research.^[16]^ In addition, because low serum CPN was part of the composite diagnostic criteria used at our center, incorporation bias cannot be excluded, and the sensitivity of CPN may be overestimated; future studies using reference standards that are independent of CPN (e.g., genetic diagnosis) are warranted.

## 5. Conclusion

This study reinforces serum CPN as a highly accurate, accessible, and clinically meaningful diagnostic marker for Wilson’s disease in children. The identified population-specific cutoff of 15 mg/dL optimizes diagnostic performance and can be readily applied in routine pediatric hepatology practice. Integrating CPN with age, sex, neurological findings, KF ring status, and urinary copper levels provides a comprehensive and practical diagnostic approach, especially in regions with limited access to genetic testing. Multicenter validation and the inclusion of emerging copper biomarkers are recommended to refine diagnostic algorithms for pediatric Wilson’s disease.

## Author contributions

**Conceptualization:** Maysam Yaldany, Fares Abboud.

**Data curation:** Fares Abboud, Sultaneh Haddad.

**Formal analysis:** Sultaneh Haddad.

**Investigation:** Maysam Yaldany, Fares Abboud.

**Methodology:** Fares Abboud.

**Validation:** Fares Abboud.

**Visualization:** Fares Abboud.

**Writing – original draft:** Maysam Yaldany, Fares Abboud, Sultaneh Haddad, Suha Madanieh, Jaber Mahmod, Manar Alhazaimeh, Ghosoun Atoom.

**Writing – review & editing:** Maysam Yaldany, Fares Abboud, Sultaneh Haddad, Suha Madanieh, Jaber Mahmod, Manar Alhazaimeh, Ghosoun Atoom.

## References

[1] Czlonkowska A Litwin T Dusek P. Wilson disease. Nat Rev Dis Primers. 2018;4:21. 30190489 10.1038/s41572-018-0018-3 PMC6416051

[2] Ferenci P Caca K Loudianos G. Diagnosis and phenotypic classification of wilson disease. Liver Int. 2003;23:139–42. 12955875 10.1034/j.1600-0676.2003.00824.x

[3] Kim JA Kim HJ Cho JM. Diagnostic value of ceruloplasmin in the diagnosis of pediatric wilson’s disease. Pediatr Gastroenterol Hepatol Nutr. 2015;18:187–92. 26473139 10.5223/pghn.2015.18.3.187 PMC4600703

[4] Cauza E Maier-Dobersberger T Polli C Kaserer K Kramer L Ferenci P. Screening for wilson’s disease in patients with liver diseases by serum ceruloplasmin. J Hepatol. 1997;27:358–62. 9288611 10.1016/s0168-8278(97)80182-1

[5] Mak CM Lam CW Tam S. Diagnostic accuracy of serum ceruloplasmin in Wilson disease: determination of sensitivity and specificity by ROC curve analysis among ATP7B-genotyped subjects. Clin Chem. 2008;54:1356–62. 18556333 10.1373/clinchem.2008.103432

[6] Merle U Eisenbach C Weiss KH Tuma S Stremmel W. Serum ceruloplasmin oxidase activity is a sensitive and highly specific diagnostic marker for wilson’s disease. J Hepatol. 2009;51:925–30. 19720421 10.1016/j.jhep.2009.06.022

[7] Xu R Jiang Y-f Zhang Y-h Yang X. The optimal threshold of serum ceruloplasmin in the diagnosis of wilson’s disease: a large hospital-based study. PLoS One. 2018;13:e0190887. 29324775 10.1371/journal.pone.0190887 PMC5764328

[8] Yang Y Hao W Wei T. Role of serum ceruloplasmin in the diagnosis of wilson’s disease: a large Chinese study. Front Neurol. 2022;13:1058642. 36570465 10.3389/fneur.2022.1058642 PMC9768184

[9] Roberts EA Schilsky ML; American Association for Study of Liver Diseases (AASLD). Diagnosis and treatment of Wilson disease: an update. Hepatology. 2008;47:2089–111. 18506894 10.1002/hep.22261

[10] Pericleous M Kelly C Schilsky ML. Diagnosis confirmation and screening of Wilson disease. In: Schilsky ML, ed. Management of Wilson Disease: A Pocket Guide. Springer; 2018:17–44.

[11] European Association for the Study of the Liver. EASL clinical practice guidelines: wilson’s disease. J Hepatol. 2012;56:671–85. 22340672 10.1016/j.jhep.2011.11.007

[12] Schilsky ML Roberts EA Bronstein JM. A multidisciplinary approach to the diagnosis and management of Wilson disease: 2022 practice guidance on wilson disease from the American association for the study of liver diseases. Hepatology. 2023;77:1428–55. 36152019 10.1002/hep.32805

[13] Ferenci P Stremmel W Czlonkowska A. Age and sex but not ATP7B genotype effectively influence the clinical phenotype of Wilson disease. Hepatology. 2019;69:1464–76. 30232804 10.1002/hep.30280

[14] Guillaud O Brunet A-S Mallet I. Relative exchangeable copper: a valuable tool for the diagnosis of Wilson disease. Liver Int. 2018;38:350–7. 28719006 10.1111/liv.13520

[15] Poujois A Trocello J-M Djebrani-Oussedik N. Exchangeable copper: a reflection of the neurological severity in wilson’s disease. Eur J Neurol. 2017;24:154–60. 27739240 10.1111/ene.13171

[16] Kasztelan-Szczerbinska B Cichoz-Lach H. Wilson’s disease: an update on the diagnostic workup and management. J Clin Med. 2021;10:5097. 34768617 10.3390/jcm10215097 PMC8584493

